# Patient Reported Outcomes (PROs) in Clinical Trials: Is ‘In-Trial’ Guidance Lacking? A Systematic Review

**DOI:** 10.1371/journal.pone.0060684

**Published:** 2013-04-01

**Authors:** Derek G. Kyte, Heather Draper, Jonathan Ives, Clive Liles, Adrian Gheorghe, Melanie Calvert

**Affiliations:** 1 Primary Care and Clinical Sciences, University of Birmingham, Birmingham, United Kingdom; 2 Medicine, Ethics, Society and History, University of Birmingham, Birmingham, United Kingdom; 3 Nursing & Physiotherapy, University of Birmingham, Birmingham, United Kingdom; 4 MRC Midland Hub for Trials Methodology Research, Birmingham, United Kingdom; Bremen Institute of Preventive Research and Social Medicine, Germany

## Abstract

**Background:**

Patient reported outcomes (PROs) are increasingly assessed in clinical trials, and guidelines are available to inform the design and reporting of such trials. However, researchers involved in PRO data collection report that specific guidance on ‘in-trial’ activity (recruitment, data collection and data inputting) and the management of ‘concerning’ PRO data (i.e., data which raises concern for the well-being of the trial participant) appears to be lacking. The purpose of this review was to determine the extent and nature of published guidelines addressing these areas.

**Methods and Findings:**

Systematic review of 1,362 articles identified 18 eligible papers containing ‘in-trial’ guidelines. Two independent authors undertook a qualitative content analysis of the selected papers. Guidelines presented in each of the articles were coded according to an *a priori* defined coding frame, which demonstrated reliability (pooled Kappa 0.86–0.97), and validity (<2% residual category coding). The majority of guidelines present were concerned with ‘pre-trial’ activities (72%), for example, outcome measure selection and study design issues, or ‘post-trial’ activities (16%) such as data analysis, reporting and interpretation. ‘In-trial’ guidelines represented 9.2% of all guidance across the papers reviewed, with content primarily focused on compliance, quality control, proxy assessment and reporting of data collection. There were no guidelines surrounding the management of concerning PRO data.

**Conclusions:**

The findings highlight there are minimal in-trial guidelines in publication regarding PRO data collection and management in clinical trials. No guidance appears to exist for researchers involved with the handling of concerning PRO data. Guidelines are needed, which support researchers to manage all PRO data appropriately and which facilitate unbiased data collection.

## Introduction

Patient reported outcomes (PROs) such as health-related quality of life (HRQL) are increasingly assessed in clinical trials.[1]–[3] PROs provide researchers, clinicians and patients with important information regarding the effect of a disease and its treatment: on symptoms (for example, pain or fatigue) and on HRQL or satisfaction with care.[4] In general, patients participating in a trial do not directly benefit from completing a PRO questionnaire. This approach is adopted to ensure trial participants are not tempted to tailor their answers in order to influence the treatment they receive within a study, which is a potential source of bias.[5], [6] PRO results are therefore used to inform the care of future patients[6], who, with their clinicians, may use PRO data to inform significant health-care decisions. For example, between interventions offering similar survival or progression-free survival rates, or those that have differing trade-offs between therapeutic benefit and undesirable side-effects.[4] Thus, it is crucial that PROs are administered and processed in an un-biased way.

In order to ensure high quality PRO trial data, consistent and rigorous standardised data collection methods should be used throughout a trial.[7] The use of standardised methods should serve to minimise errors, measurement variability, missing data and systematic bias, thus contributing to the validity of trial results.[8] Local site staff require access to ‘in-trial’ (i.e. recruitment, data collection and data inputting, see Box S1) guidelines that clearly outline the standardised methods in-use, so that all study personnel may fully incorporate them into practice. Such guidelines should be contained within the trial protocol, supported by standard operating procedures (SOPs) where appropriate.

It is of concern, therefore, that anecdotal evidence - obtained during national quality of life training days run by the MRC Midland Hub for Trials Methodology in the UK - suggests that in-trial PRO guidelines are not routinely included within trial documentation and that, as a result, unstandardised PRO data collection may be common. Researchers also report feeling particularly uncomfortable that they receive no specific guidance on how to manage ‘concerning’ PRO data, i.e. data that might raise concern for the wellbeing of the trial participant in some way. Staff encountering such data - commonly represented by markedly low HRQL scores, or unexpected unprompted additional information recorded on the back of questionnaires - were therefore unsure where their responsibility should lie, or whether they should be viewing this information in the first place. In this situation, some described experiencing a ‘dual-role’ tension between their concurrent responsibilities as a clinician and researcher: the duty to act upon the information to benefit the patient verses that of protecting trial integrity by not intervening. In some instances, reports indicated that off-protocol concomitant interventions had been administered, some of which may not have been captured by standard trial reporting mechanisms. Such interventions have the potential to bias trial results. These anecdotal reports have since been supported by a recently completed qualitative study, in which we used semi-structured interviews to explore the experiences of 26 research nurses, research facilitators, trial coordinators and data managers across three NHS sites and two clinical trials units in the UK[9] (under review). This study confirmed a potential for bias associated with concerning PRO data, during both postal or clinic-based and self-reported or researcher/research nurse-assisted data collection.

These reports suggest a lack of in-trial PRO guidance, with a subsequent absence of systematic monitoring of potentially concerning PRO data and a resulting risk of bias. It is uncertain, however, whether they also reflect a deficiency in the published literature in this area. There are recent publications concerning the design of trials with a PRO outcome[7], [10] and, with the development of the CONSORT PRO extension[11], there is now guidance to improve PRO reporting: it remains unclear if the literature provides adequate coverage of in-trial issues.

The purpose of this study was to systematically review the current published in-trial PRO guidance, as no review of this kind had been previously undertaken. The objectives for our review were:

- To investigate the extent and content of the current in-trial PRO guidelines in publication.
- To determine if these guidelines adequately address questions raised by researchers involved in PRO data collection, surrounding the management of concerning PRO data.

## Methods

### Search strategy

The MEDLINE (Ovid), EMBASE, AMED and CINHAL+ databases were searched from inception to March 2012 (electronic search strategies are presented in full in Appendix S1). We also searched; the US Food and Drug Administration[12], European Medicines Agency[13], General Medical Council[14], Medical Research Council[15] and Royal College of Nursing[16] websites; PROQUEST (Thesis repository); Google; and made use of expert communication in an attempt to find additional potentially eligible papers not returned during the electronic database search. Records were first screened by title/abstract before full-text articles were retrieved for eligibility evaluation. Remaining articles were then subject to a citation search before a final hand-search of all reference lists.

### Identification of eligible studies

Papers were deemed eligible if they included any form of in-trial guideline focused on PRO assessment during clinical trials. We defined the term ‘in-trial’ as relating to recruitment, data collection and data inputting activity, occurring from the first participant recruitment, through to inputting the final participant's data. The reviewers used the Oxford English Dictionary definition of the word ‘guideline’ during eligibility screening; “a general rule, principle, or piece of advice”.[17] Non-English papers were excluded. There were no other restrictions. All citations were downloaded into Endnote® software version 14, and duplicates deleted. DK screened all articles by title/abstract to determine their eligibility and AG reviewed a random sample of 10% in order to evaluate the reliability of the selection process. Agreement was high (Kappa = 0.903) and any discrepancies were resolved through discussion. Full text articles were retrieved following first round exclusions and were also subject to two independent eligibility reviews (DK 100%, AG 10%), this time with perfect agreement.

### Data extraction

Data extraction occurred following the final selection of included articles.

DK and CL independently searched each paper to identify all sentences that provided any type of ‘guideline statement’ (which we defined as ‘an expression in words of a general rule, principle, or piece of advice') regarding PRO measurement (in-trial or otherwise). A consensus meeting was then held, to resolve any disagreements and finalise the selection. Each sentence, representing one ‘guideline statement’, was then extracted, as a text excerpt, into a mixed-method data analysis software package (Dedoose © 2011 SCRC) and tagged with its source data (Article title, Journal, Year of publication).

### Data analysis

**Figure 1:**
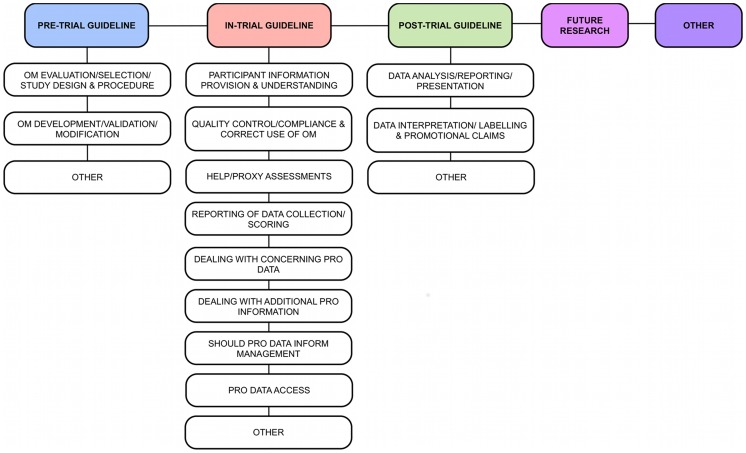
Definitive coding frame. Major categories in bold.

DK and CL undertook a qualitative content analysis[18] of the excerpts extracted from the included papers. All text excerpts were categorised according to an *a priori* coding frame, which was developed using a concept-driven strategy (i.e. codes were assigned based on the authors' prior knowledge of the literature and the study research questions). DK and CL piloted the coding framework, each independently applying the first draft to a random selection of the included papers[6], [7], [19] (n = 3 (17%)). Following the pilot, a meeting was held to discuss issues requiring clarification and to reach consensus regarding the data-driven changes that would improve the validity of the framework. Three of the co-authors (MC, HD and JI), who possess expertise in PRO design, implementation, reporting and ethics, checked and approved the face validity of the final coding frame. The definitive coding frame is presented in Figure 1. During the main analysis, DK and CL independently categorised each guideline statement according to the phase of trial activity to which it pertained, using a major dimension within the coding frame. These major dimensions were as follows; ‘Pre-Trial’, which included all content relating to the trial inception (including training logistics), up to the start of recruitment; ‘In-Trial’, denoting content directly related to the act of trial recruitment, data collection and inputting; ‘Post-Trial’, including activity taking place following data collection, for example, data analysis/reporting; ‘Future Research’, representing statements addressing the future direction of PRO research activity; and ‘Other’, used to identify guideline statements not captured in the main coding categories. Each individual guideline was also sub-categorised, as appropriate, in order to further identify its role within a given area.

Throughout both the pilot and the main analysis phase, the reviewers met frequently to determine coding reliability for each paper and to seek consensus regarding coding disagreements. The reliability of coding application was determined using Cohen's kappa statistic.[20] Specifically, pooled kappa was employed, as it is the preferred method of calculating inter-rater agreement across a large number of coding items.[21] Face validity of the coding frame was further evaluated by determining the proportion of codes applied to the residuals (i.e., the ‘Other’ major- and sub-categories). A high level of residual coding may indicate that the main categories of the coding frame do not adequately describe the concept under study.[18] Whilst there are no firm guidelines regarding the desirable level of residual coding, we theorised that a figure of less than 5% would support the validity of our coding frame.

A protocol was not published or registered for this study. However, all reviewers followed a protocol detailing *a priori* determined search strategies, data extraction and data analysis methods.

## Results

### Included studies

**Figure 2:**
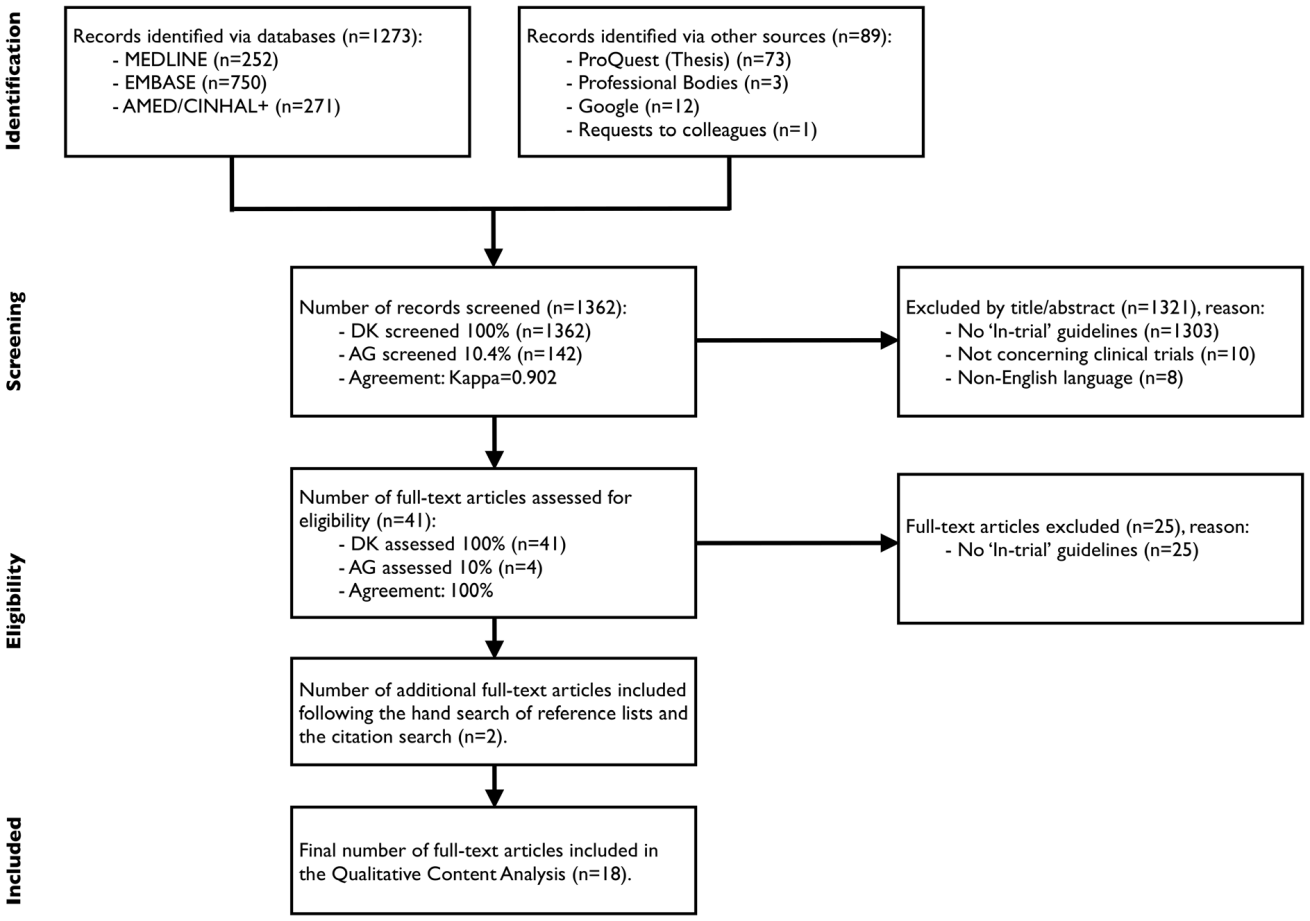
PRISMA flow diagram of study selection process.

The search strategy yielded 1273 citations from MEDLINE, EMBASE, AMED and CINHAL+, 89 citations were returned using other sources (PROQUEST, professional bodies, Google, expert communication) (PRISMA[22] flow diagram, Figure 2). In total, 41 full text articles were retrieved for review. 25 articles were excluded at this stage, as they contained no in-trial guideline statements. An additional 2 papers were included following the reference list and citation searches. A final total of 18 relevant articles were included in the analysis.

### Study characteristics

The characteristics of the 18 included papers are summarised in Table 1. The majority of papers were concerned with the incorporation of PRO/HRQL measures into cancer trial design.[5], [6], [10], [23]–[28] Several considered PRO issues relating to pharmaceutical prescribing/labelling.[1], [7], [29]–[32] Two papers presented generalised guidance on using PRO/HRQL measures in clinical trials.[33], [34] Finally, one paper presented recommendations for PRO/HRQL assessment in allergy-related clinical trials.[19] The included articles were drawn from 16 different sources and the mean number of excerpts extracted from each paper was 58 (range 16–127).

**Table 1:** Study characteristics.

Included Studies	Year of Publication	Source	Excerpts Extracted
Baiardini et al [19]	2010	Allergy	46
Basch et al [10]	2011	Value in Health	120
Calvert & Freemantle [1]	2004	Journal of Clinical Pharmacy and Therapeutics	74
Chassanay et al [29]	2002	Drug Information Journal	127
FDA [30]	2006	Health and Quality of Life Outcomes	86
FDA [7]	2009	FDA Website	116
Fayers [5]	1995	Quality of Life Research	18
Fayers et al [6]	1997	European Journal of cancer	62
Fletcher [23]	1995	British Journal of Clinical Pharmacology	48
Fletcher et al [24]	1992	BMJ	34
Hopwood et al [25]	1997	European Journal of cancer	25
Kiebert et al [26]	1998	Statistics in Medicine	16
Leidy et al [31]	1999	Value in Health	89
Luo & Cappelleri [33]	2008	Clinical Research and Regulatory Affairs	63
Moinpour et al [27]	1989	Journal of the National Cancer Institute	57
Movsas [28]	2003	Seminars in Radiation Oncology	39
Poulter [34]	1997	Good Clinical Practice Journal	19
Revicki et al [32]	2000	Quality of Life Research	80

### Data synthesis

Over 1,110 guideline statements were extracted and coded following review of the 18 papers. The coding frame demonstrated reliability, with pooled kappa ranging from 0.86 to 0.97 across articles, and face validity, with overall residual coding at 1.2%. A summary of the final coding breakdown is presented in Table 2.

**Table 2:** Coding summary.

Coding Categories	Example Excerpts
‘IN-TRIAL’ GUIDELINES (9.2%)	
Quality control, compliance & correct use of OM (61.2%)	“In order to maximize compliance when administering the questionnaire investigators should… check the questionnaire for completeness at the time of visit and prompt patients to try and complete any missing items.” [1]
Help/proxy assessments (16.5%)	“Interviewers and proxies should be consistent during the trial.” [29]
Reporting of data collection/scoring (9.7%)	“The reasons for missing data should be recorded at the time of occurrence and later considered to lend insight into the potential patterns for why data are missing.” [33]
Participant information provision & understanding (7.8%)	“The patient must fully understand the purpose of the QOL assessments.” [34]
Should PRO data inform management (4.8%)	“Not only, therefore, should the information… not be used to influence treatment, but the patient should be informed clearly that their replies are confidential…” [5]
‘PRE-TRIAL’ GUIDELINES (9.2%)	
OM evaluation, OM selection, study design & procedure (87%)	“Protocols should include clear justification for the assessment of HRQL, provide details of the instrument and its properties, specify timings of assessments and emphasize the need to maximize compliance.” [1]
OM development, validation, modification (12.8%)	“A PROs tool can only be used in a language that differs from the original after translation and back-translation, and a cross-cultural validation is performed.” [19]
Other (0.2%)	“Requests for FDA input should be addressed to the review division responsible for the medical product…” [7]
‘POST-TRIAL’ GUIDELINES (15.8%)	
Data analysis, reporting, presentation (67.7%)	“In settings where there is a large proportion of missing data due to toxicity, morbidity or mortality, sensitivity analysis should be performed to address the possibility that the missing data are non-ignorable or not missing at random.” [32]
Data interpretation, labeling & promotional claims (33.3%)	“We suggest that, in general, two well-designed randomized clinical trials with unequivocal results should provide sufficient evidence of an HRQL effect to substantiate a claim in a given population.” [31]
‘FUTURE RESEARCH’ (1.8%)	“A need exists to standardise the terminology used in studies and to define a minimum set of concepts and dimensions of quality of life in order to justify a claim to have measured quality of life.” [23]
‘OTHER’ (1%)	“We encourage instrument developers to make their instruments and related development history available and accessible publicly.” [7]

Major coding categories in bold. Abbreviations - OM: Outcome measure, QOL: Quality of Life, HRQL: Health-Related Quality of Life, PRO: Patient-Reported Outcome, FDA: Food & Drug Administration.

### Major coding categories

‘In-trial’ guidance, whilst present in all papers, did not represent the major focus of any, accounting for 9.2% of guideline content across the articles reviewed. ‘Pre-trial’ guidelines were predominant throughout (72.2%), again present in all papers. ‘Post-trial’ guidance was the next most prevalent category (15.8%), presented across 13 articles.[1], [5], [7], [10], [19], [23], [24], [28]–[33] Statements pertaining to ‘future research’ represented 1.8% of guidelines (9 papers)[10], [19], [23], [24], [26]–[28], [30], [31] and the major category ‘Other’ was attributed to 1% of content (8 papers).[7], [10], [24], [26], [27], [29]–[31]

### Sub-categories

#### In-trial

There were no guideline statements addressing the management of concerning PRO data, or related questions including how additional information recorded on the back of questionnaires should be handled and who should have routine access to PRO data in the first instance. The majority of in-trial guidelines (61.2%) tackled notions surrounding quality control, compliance and the correct use of PROs.[1], [5]–[7], [10], [19], [24]–[31], [33], [34] Authors highlighted the importance of minimising missing items during data collection.[6], [34] A number of papers presented guidance aimed at improving compliance within a trial in order to maximise data quality: examples included the proposed education of local site staff, training of patients and use of real-time adherence monitoring [1], [5], [6], [10], [24], [25], [27]–[29], [34]. Other guidelines were concerned with piloting[27] and standardisation[28], [31] of data collection. Examples of suggested methods of standardisation included the following:

- A named individual, concerned with quality control, serving as a PRO data collection contact at each research site within a trial.[1], [6], [10]
- The use of standard scripts in interview- or telephone-based questionnaires.[31]
- Ensuring that patients complete questionnaires at the same pre-specified time point, usually selected so as to avoid the undue influence of a preceding event.[31], [33]

Where a trial participant is unable to complete their PRO questionnaire, a proxy (commonly a partner or close relative) may be asked to complete the form on their behalf. Discussion surrounding the role of proxies represented 16.5% of in-trial guidelines.[1], [5], [6], [23], [29], [31], [32] Authors mainly highlighted the situations in which proxy assessment was justified.[1], [5], [6], [29], [31], [32] The use of a proxy was generally promoted as a last resort [1], [6], [29], however it was acknowledged that proxy data was better than no data at all.[5], [29] The ideal identity of the proxy was discussed by two authors, who concluded that, if possible, the same person should be used throughout the trial[29] and they should be close enough to the patient to provide valid data.[32] Guidelines for the reporting of data collection represented 9.7% of in-trial content[5], [6], [10], [26], [29], [33] and were primarily concerned with the need to document reasons for non-compliance[5], [6], [10], [33] and the need to report whether or not a proxy was used[6], [29]. A small number of in-trial guideline statements (7.8%) focused on patient information, endorsing the use of a supplementary leaflet for patients to take home[6], and highlighting the importance of the investigator in ensuring the patient fully understands the role of PRO measurement.[34] Two papers by the same author[5], [6] presented guidelines suggesting that PRO data should not be used to influence management during a trial and one paper suggested that trial participants ought to be informed when data would be used for the benefit of future patients only.[10]

#### Pre-trial

The majority of pre-trial guidelines (87%) were focused on study design, procedural issues (including training logistics) and the evaluation/selection of appropriate PRO measures.[1], [5]–[7], [10], [19], [23]–[34] Others (12.8%) were concerned with questionnaire development and validation, or with issues arising from questionnaire modification.[1], [7], [10], [19], [23], [24], [28]–[33]

#### Post-trial

Most post-trial guidelines (66.7%) concentrated on data analysis, reporting and presentation issues.[1], [5], [7], [10], [19], [23], [24], [28]–[33] The remaining guidance in this area (33.3%) surrounded the interpretation of PRO data and related labeling claims.[5], [7], [19], [23], [24], [28]–[33]

## Discussion

The purpose of this review was to investigate whether anecdotal claims (subsequently confirmed by data under review), highlighting a lack of in-trial PRO guidance, reflect a deficiency in the published literature in this area. Our main findings suggest there a minimal guidelines in publication focused on in-trial PRO activity and there are a complete lack of guidelines addressing the management of concerning PRO data.

Of the small number of in-trial guidelines that are in circulation, the majority appear to deal with the procedural issues associated with the prevention of missing data. This focus may be understandable given the detrimental effect missing data may have on a trial. Trial reports indicate that PRO questionnaires are commonly returned with incomplete entries and some may not be returned at all.[7] This data may not be missing at random and it represents a serious potential bias when present.[10] Therefore, it is encouraging there is some consensus in the guidelines reviewed. To reduce missing PRO data, authors recommended that:

- The investigator/research nurse should: (1) motivate the patients to complete all questionnaires in-full by ensuring they understand the purpose and importance of the PRO assessment within the trial, (2) check questionnaires for completeness and prompt patients to fill in any missing items, (3) show appreciation for the efforts of the patient in completing the questionnaire.[1], [5], [6], [25], [27]–[29], [33], [34]
- PRO data is best collected in clinic, in an environment that is private and free from distraction.[1], [24], [29], [34]
- A centrally managed PRO data monitoring system should be in place, coordinated at each site by a named individual, tasked with; evaluating compliance across trial locations, issuing data collection reminders to patients where needed and chasing-up missing items.[1], [6], [10], [25], [27], [28]

The guidance surrounding missing data is therefore comprehensive. In contrast, no guidelines appear to adequately address aspects surrounding the management of concerning PRO data. This may be a problem given this issue has been identified as key by those involved in PRO data collection, as it can result in dual-role tension and may risk the potential introduction of bias into a trial.

A PRO questionnaire may be the only outcome within a trial capable of identifying ‘tolerable’ symptoms such as participant anxiety or depression; and the research nurse checking the form may be the only individual to whom participants have disclosed how they feel. Understandably, nurses may feel it is their duty to intervene when faced with PRO data that raises concern for the participant. A problem arises if the intervention is non-medicinal; for example, words of comfort, or advice to visit one's general practitioner, or if the advice results in the participant self-medicating. Direct medicinal interventions are far more easily controlled-for during data analysis. Non-medicinal or self-directed interventions, that are selectively delivered in response to concerning PRO data, may influence patient well-being but remain unrecorded in the trial documentation: this may represent a hitherto unforeseen source of bias.

Research nurses have reported experiencing dual-role tension when handling PRO data. Dual-role tension arises when an individual's values and responsibilities as a researcher conflict with those associated with being a clinical practitioner. Assuming ethical norms have been followed and participant ‘risk and burden’ does not outweigh the potential benefit of trial participation [35], the nurse *researcher* may justifiably choose not to intervene when concerning PRO data is disclosed, in order to protect trial integrity. This decision may be driven by consequentialist values, geared toward achieving the greatest benefit at the lowest cost, and reasoning that the benefits of producing unbiased trial results outweigh the personal costs experienced by the ‘few’ participants who continue to (tolerably) suffer. Conversely, nurse *practitioners* are obliged to make the care of their patients their first concern, as outlined in the Nursing and Midwifery Council code of conduct[36], which compels them to take steps to address any evident suffering. This conflict between the two professional duties has been recognized elsewhere[37]–[39]. However, what sets PRO data collection apart from the management of other trial outcomes is the current lack of published, and trial-based, guidance in this area. In our experience, the trial protocol often contains clear guidelines surrounding the levels at which some clinical outcomes, blood pressure for example, need to reach before the data collector should become concerned.[9] There is usually also a clear system in place to manage participants whose clinical measurements exceed agreed limits. Equivalent guidance is not always provided for PROs. Thus, the researcher collecting/inputting PRO data may be left to determine independently, on a case-by-case basis, whether PRO results signal a risk to the participant that outweighs the benefit of trial involvement. We believe this situation places unreasonable demands upon the researcher and promotes inconsistency, as there is unlikely to be uniformity in decision-making across trial sites; this may adversely affect data quality. Our findings highlight the need to develop and publish specific guidelines that clearly outline how concerning PRO data should be handled, as there are none currently in circulation. PRO in-trial guidelines should be brought in line with those covering traditional clinical outcomes and should define the conditions under which the researcher may take remedial action, and the form this intervention might take.

### Limitations

Non-English language papers were excluded from the review, which potentially lessens the generalisability of the results presented. However, this decision was taken as a key element of qualitative content analysis involved determining the implied or latent meaning of the material.[18] We questioned the validity of such analysis using material translated from the original language by a third party, as some latent meaning may be lost during the translation process. Our search strategy dictated that we carefully reviewed papers for their guideline content only if their title/abstract gave an indication that some aspect of in-trial activity might be discussed. It is possible that papers providing ‘in-trial’ guidance exist, which make no reference to in-trial activity in their title or abstract.

## Conclusions

In-trial guidelines aimed at PRO recruitment, data collection and data inputting within clinical trials are lacking. No guidance appears to exist for researchers involved with the handling of concerning PRO data. This is a worry as this activity may be associated with considerable personal and professional anxiety and may risk the introduction of bias when the ethical tension generated, is resolved in favour of responding to the needs of the patient over the expectations of the trial. Further research is needed to produce guidelines aimed at supporting researchers so they can deal effectively with dual-role tensions, manage PRO data appropriately and facilitate unbiased data collection.

## Supporting Information

Appendix S1 **Search strategies.**
(DOCX)

Box S1 **Definition of terms.**
(DOCX)
