# Correction: Patient Reported Outcomes (PROs) in Clinical Trials: Is ‘In-Trial’ Guidance Lacking? A Systematic Review

**DOI:** 10.1371/annotation/e7d2b920-2c2a-425e-b500-982cd72f4d64

**Published:** 2014-01-02

**Authors:** Derek G. Kyte, Heather Draper, Jonathan Ives, Clive Liles, Adrian Gheorghe, Melanie Calvert

In Table 2, the first column, seventh row should read "PRE-TRIAL Guidelines (72.2%)".

