# Supplementary material for: Patient Reported Outcomes (PROs) in Clinical Trials: Is ‘In-Trial’ Guidance Lacking? A Systematic Review
Source: PLoS One. 2013 Apr 1;8(4):e60684. doi: 10.1371/journal.pone.0060684 (PMC3613381; doi:10.1371/journal.pone.0060684)
Supplement: Appendix S1 — Search strategies. (DOCX) [file pone.0060684.s001.docx]

### Appendix I

**Search Strategies**

# MEDLINE

1 - "Patient reported outcome*".ti.

2 - "Patient-reported outcome*".ti.

3 - "Health-related quality of life".ti.

4 - "Health related quality of life".ti.

5 - "Quality of Life".ti.

6 - *"Quality of Life"/

7 - 1 or 2 or 3 or 4 or 5 or 6

8 - exp Guideline/ or exp Practice Guideline/

9 - *Health Policy/

10 - (guideline* or Guide or Guidance or Recommendations or Standards).m_titl.

11 - 8 or 9 or 10

12 - 7 and 11

# AMED/CINHAL+ (EBSCO)

S1 - TI Patient reported outcome*

S2 - TI patient-reported outcome*

S3 - TI Health related quality of life

S4 - TI Health-related quality of life

S5 - TI quality of life

S6 - S1 or S2 or S3 or S4 or S5

S7 - Guideline* or Practice Guideline*

S8 - Health policy

S9 - TI Guide or Guidance or Recommendations or Standards

S10 - S7 or S8 or S9

S11 -S6 and S10

EMBASE

1 - "Patient reported outcome*".ti.

2 - "Patient-reported outcome*".ti.

3 - "Health-related quality of life".ti.

4 - "Health related quality of life".ti.

5 - "Quality of Life".ti.

6 - *"Quality of Life"/

7 - 1 or 2 or 3 or 4 or 5 or 6

8 - exp Guideline/ or exp Practice Guideline/

9 - *Health Policy/

10 - (guideline* or Guide or Guidance or Recommendations or Standards).m_titl.

11 - 8 or 9 or 10

12 - 7 and 11
