# Supplementary material for: Patient Reported Outcomes (PROs) in Clinical Trials: Is ‘In-Trial’ Guidance Lacking? A Systematic Review
Source: PLoS One. 2013 Apr 1;8(4):e60684. doi: 10.1371/journal.pone.0060684 (PMC3613381; doi:10.1371/journal.pone.0060684)
Supplement: Box S1 — Definition of terms. (DOCX) [file pone.0060684.s002.docx]

**Box 1**. Definition of Terms.

| Patient-Reported Outcome (PRO) | A health status report that comes directly from the patient[[7](#_ENREF_7)] |
| --- | --- |
| Health-Related Quality of Life (HRQL) | The effect of a disease and its treatment on an individuals physical, emotional and social well-being[[4](#_ENREF_4)] |
| In-Trial Activity | Activity spanning trial recruitment, data collection and data inputting |
| Concerning PRO Data | PRO data that raises concern for the wellbeing of the trial participant |
| Proxy PRO Assessment | Where an individual with a comprehensive knowledge of the trial participant is asked to complete the PRO on their behalf |
